# Glycyrrhetinic acid regulates impaired macrophage autophagic flux in the treatment of non-alcoholic fatty liver disease

**DOI:** 10.3389/fimmu.2022.959495

**Published:** 2022-07-28

**Authors:** Yadong Fan, Wenjin Dong, Ying Wang, Shan Zhu, Rundong Chai, Zhe Xu, Xiaoyu Zhang, Yiqi Yan, Long Yang, Yuhong Bian

**Affiliations:** ^1^School of Integrative Medicine, Tianjin University of Traditional Chinese Medicine, Tianjin, China; ^2^Department of Science and Education, Tianjin Union Medical Center, Tianjin, China; ^3^State Key Laboratory of Component Traditional Chinese Medicine, Tianjin University of Traditional Chinese Medicine, Tianjin, China; ^4^The Reproductive Center, First Teaching Hospital of Tianjin University of Traditional Chinese Medicine, Tianjin, China; ^5^Research Center for Infectious Diseases, Tianjin University of Traditional Chinese Medicine, Tianjin, China

**Keywords:** glycyrrhetinic acid, nonalcoholic fatty liver disease, macrophage, autophagic flux, STAT3-HIF-1α pathway

## Abstract

Macrophages are involved in hepatocyte steatosis and necroinflammation and play an important role in the pathogenesis of non-alcoholic fatty liver disease (NAFLD). Impaired autophagy function (decreased autophagy or blocked autophagic flow) leads to cell damage and death and promotes NAFLD progression. The experimental and clinical research of glycyrrhetinic acid (GA) in the treatment of NAFLD has gradually attracted attention with clear pharmacological activities such as immune regulation, antiviral, antitumor, antioxidant, liver protection, and anti-inflammatory. However, the effects of GA on the STAT3-HIF-1α pathway and autophagy in macrophages are still unclear, and its mechanism of action in the treatment of NAFLD remains to be further elucidated. We constructed a NAFLD mouse model through a high-fat and high-sugar diet to investigate the therapeutic effects of GA. The results showed that GA reduced weight, improved the pathological changes and hepatic lipid deposition of liver, and abnormally elevated the levels of serum biochemical (AST, ALT, TG, T-CHO, LDL-C, and HDL-C) and inflammatory indexes (IL-1β, IL-4, IL-6, MCP-1, and TNF-α) in NAFLD mice. Further examination revealed that GA ameliorates excessive hepatic macrophage infiltration and hepatocyte apoptosis. The results of the cell experiments further elaborated that GA modulated the PA-induced macrophage STAT3-HIF-1α pathway and ameliorated impaired autophagic flux (blockade of autophagosome–lysosome fusion) and overactivation of inflammation. Excessive hepatocyte apoptosis caused by the uncontrolled release of inflammatory cytokines was also suppressed by GA.

**Conclusion:**

This study demonstrated that GA could regulate the STAT3-HIF-1α pathway of macrophages, ameliorate the impaired autophagy flux, and reduce the excessive production of inflammatory cytokines to improve the excessive apoptosis of liver cells, thus playing a therapeutic role on NAFLD.

## Introduction

With changes in dietary patterns and lifestyles, a global pandemic of chronic metabolic diseases has emerged. In this context, non-alcoholic fatty liver disease (NAFLD) is a metabolic stress-induced liver injury closely related to genetic susceptibility and insulin resistance, whose incidence is increasing year by year. According to statistics, the global incidence of NAFLD is about 25% (1), and the prevalence in China is about 29.2% (2), accompanied by an increasing trend of younger age (3). Metabolic syndromes (Mets), especially hypertension, type 2 diabetes, cardiovascular disease, and obesity, are closely related to the pathophysiological process of NAFLD (4, 5). The pathogenesis of NAFLD is complex, with theories such as “two-hit” and “multi-hit”. Lipid metabolism imbalance, immune system imbalance, inflammasome activation, mitochondrial dysfunction, endoplasmic reticulum stress, and intestinal microbial disorders can affect the pathogenesis and progression of NAFLD (6). The high concealment of NAFLD, multisystem involvement, and heavy medical expenses have caused serious social medical burden (7).

In NAFLD, free fatty acids (FFAs) are important mediators of lipotoxicity, which lead to cellular damage through different pathways including endoplasmic reticulum stress (8). This process is balanced by the upregulation of autophagy pathways that maintain cell survival and homeostasis. Impaired autophagy is a key factor in hepatocyte injury in NAFLD (9). Macrophages are involved in hepatocyte steatosis and necroinflammation and play an important role in the pathogenesis of NAFLD. Excessive infiltration of macrophages and overproduction of proinflammatory cytokines in the liver contribute to the pathogenesis of NAFLD (10). STAT3 has been well regarded as a key regulatory target of the development and progression in liver inflammation, injury, regeneration, activation of hepatic stellate cells, and liver cancer (11–15). As a key regulator of hypoxia, HIF-1α can broadly regulate the expression of hypoxia-inducible genes and the activation of various signaling pathways (16, 17). In the early stage of a high-fat diet and obesity, high levels of HIF-1α expression and hypoxia in white adipose tissue occur before the onset of insulin resistance and inflammation (18). The study confirms that the HIF gene in the liver is significantly elevated in NAFLD. The activation of HIF-1α in the liver and hepatocytes can promote lipid accumulation and liver damage to accelerate disease progression in a methionine–choline-deficient diet-induced NAFLD mouse (19). Clinical studies have also shown that HIF-1α is functionally activated in children with NAFLD accompanied by nocturnal hypoxia, which induces the expression of key genes of epithelial–mesenchymal transition and accelerates the progression of NAFLD (20). A recent study shows that HIF-1α-mediated autophagy injury in macrophages increases IL-1β production, which contributes to choline-deficient diet-induced NAFLD. STAT3 signaling is considered an upstream regulator of HIF-1α (21), and the activated STAT3 combines with the HIF-1α promoter and induces increasing HIF-1α expression. Silencing STAT3 will block HIF-1α expression even under hypoxic conditions (22). In the pathogenesis and progression of NAFLD, the STAT3-HIF-1α pathway plays a key role and deserves further exploration.

At present, there is no clinically approved specific drug for NAFLD. Glycyrrhizic acid (GL) and glycyrrhetinic acid (GA) are extracted from the roots of *Glycyrrhiza uralensis* Fisch. It has immunomodulatory, antiviral, antitumor, antioxidant, liver protection, and anti-inflammatory effects (23). GL can regulate hepatocyte lipid metabolism, glucose homeostasis, and insulin sensitivity in NAFLD mice (24–26). GL also restores bile acid homeostasis in NAFLD mice and inhibits liver inflammatory injury (27). In addition, GA restores the retinol metabolic balance in NAFLD mice to achieve therapeutic purposes (28). The early result shows that GA can prevent hepatic failure induced by lipopolysaccharide/D-galactosamine, reduce mortality and alanine aminotransferase (ALT)/aspartate aminotransferase (AST) elevation, and improve liver pathological injury. Its mechanism is related to the upregulation of macrophage interleukin-1 receptor-associated kinases M, deactivation of NF-κB, and inhibition of TNF-α production (29). However, the effect of GA on the STAT3-HIF-1α pathway and autophagy in macrophages is still unclear, and its mechanism of action in the treatment of NAFLD remains to be further elucidated. We aimed to investigate whether GA could ameliorate high-fat and high-sugar diet-induced NAFLD by modulating macrophage autophagic flux through the STAT3-HIF-1α pathway.

## Materials and methods

### Experimental animals

A total of 24 male C57BL/6 mice (SPF; age 8 weeks; weight, 19.2 ± 1.2 g) were purchased from Beijing Weitong Lihua Laboratory Animal Technology Co., Ltd., and housed in the Laboratory Animal Center of Tianjin University of Traditional Chinese Medicine (animal license number: SCXK (Beijing) 2019-0009). The animals were maintained under standard conditions of temperature (22 ± 2°C) and humidity (50 ± 5%) in a 12-h light/dark cycle. Animals were allowed free access to food and water throughout the experimental period. The operation and feeding procedures of the experimental animals abided by the relevant regulations of the Tianjin University of Traditional Chinese Medicine on the feeding and use of experimental animals.

### Establishment of the NAFLD mouse model

After 1 week of adaptive feeding, mice were randomly divided into different experimental groups. Normal mice were fed with standard chow for 12 weeks. Mice in the NAFLD model group were fed a NAFLD/NASH high-fat-rich diet and drinking water containing fructose (with 55% of fructose and 45% sucrose by weight, 42 g/l of carbohydrates was mixed in drinking water, filter sterilized) for 12 weeks (30–32). On the basis of the model group, the experimental group was administered α-GA (Lot: 20170520, Tianjin Zhongyi Pharmaceutical Co., Ltd.) by intragastric administration (60 mg/kg) for 2 weeks. The intragastric administration volume was 0.01 ml/g, and the drug was dissolved by ultrasound with 0.5% sodium carboxymethylcellulose. The mice of the normal group and the model group were gavaged with co-solvent.

### Cell treatments

Cells were purchased from the ATCC Cell Bank. The RAW264.7 cell culture condition was 10% fetal bovine serum (FBS) + 90% DMEM. The Kupffer cell culture condition was 10% FBS + 90% RPMI-1640. The AML-12 cell culture condition was 10% FBS + 90% DMEM/F12 + 10 µg/ml insulin + 5.5 µg/ml transferrin + 5 ng/ml selenium + 40 ng/ml dexamethasone. Cells were cultured in an incubator at 37°C with 5% CO_2_ concentration. Trypsin (0.25%) was used for digestion and passage. For experimental design, cells with 80%–90% cell fusion degree during the growth period in good condition were taken. Before the experiment, the CCK8 method was used to investigate the effects of palmitic acid (PA) (Lot: P0500, Sigma) coupled with bovine serum protein (without fatty acids) (Lot: A8850, Beijing Solaibao Technology Co., Ltd.) and α-GA on macrophage activity. In selected studies, cells were treated with the autophagy inhibitor 3-MA (5 mM, Lot: M833793, Macklin) for 30 min prior to PA and/or α-GA treatment, or bafilomycin (100 nM, Lot: A8510, Beijing Solaibao Technology Co., Ltd.) for the last 2 h of the PA and/or α-GA treatment. The mechanism study using the STAT3 small-molecule inhibitor (Stattic, 10 μm, Lot: S7024, Shanghai Selleck Biotechnology Co., Ltd.) and HIF-1α inhibitor Lificiguat (YC-1, 20 μM, Lot: S7958, Shanghai Selleck Biotechnology Co., Ltd.) complied with PA and/or α-GA treatment. CCK8 experiments were carried out in 96-well cell culture plates at a density of 5 × 10 (3) cells per well. The cells were seeded in a 12-well cell culture plate at a density of 1 × 10 (5) cells per well to design other experiments. After the cells were plated, the cells were cultured in an incubator for 24 h before intervention, and three replicate wells were set up in each group.

### Hematoxylin–eosin and Oil Red O staining of the liver

During the experiment, the general state of the mice in each group was observed, and the body weight of the mice was recorded every week. After the last intragastric administration, the mice were fasted for 12 h and then weighed. After 15 weeks, the mice were anesthetized with 0.3% sodium pentobarbital solution, and the liver, thymus, and spleen of the mice were taken out. The organs were rinsed with normal saline and dried with filter paper to calculate the organ index. The same part of the liver of the mice in each group with a size of 2 cm (3) was excised. After fixation in 4% paraformaldehyde solution for 72 h, the tissues were cut into 5-μm slices and stained according to the standard hematoxylin–eosin (H&E) procedure. Another part of the liver tissue was prepared into frozen sections with a thickness of 10 μm. The prepared Oil Red O working solution (Oil Red O storage solution: distilled water = 3:2) was poured into the dye vat, the sections were submerged upright, stained for 30 min, and the excess dye was washed away with 60% isopropanol. The sections were rinsed three times with distilled water, differentiated with 75% ethanol, terminated with tap water, stained with hematoxylin for 90 s, and then mounted after returning to blue. All sections were photographed and examined microscopically.

### Serum biochemical analysis

Before the mice were sacrificed, the blood of the mice was collected and serum samples were prepared. The biochemical indicators such as ALT, AST, high-density lipoprotein cholesterol (HDL-C), low-density lipoprotein cholesterol (LDL-C), total cholesterol (T-CHO), triglyceride (TG), creatinine (CRE), and blood urea nitrogen (BUN) were detected using an automatic biochemical analyzer (Microlab 300 from Rittal, the Netherlands). According to the instructions of the reagent manufacturer (Lot: 70-EK201B/3; 70-EK204/2; 70-EK2062/2; 70-EK206; 70-EK2822/2, Hangzhou Lianke Biotechnology Co., Ltd.), ELISA detection reagents of interleukin 1β (IL-1β), interleukin 4 (IL-4), interleukin 6 (IL-6), monocyte chemoattractant protein 1 (MCP-1), and tumor necrosis factor α (TNF-α) were used to detect their serum and cellular supernatant levels respectively, and absorbance at 450 nm was measured using a microplate spectrophotometer (Thermo Fisher Varioskan Flash). Before the experiment, the serum and supernatant in each group were thoroughly shaken and mixed.

### Observation of macrophage infiltration in the liver

The paraffin sections were stained according to the immunohistochemical detection kit procedure (Lot: PK10006, Proteintech Group, Inc., China): deparaffinization to water, antigen heat retrieval (citric acid retrieval solution), endogenous peroxidase inactivation, 5% goat serum blocking, F4/80 antibody (1:200, Lot: 70076S, Cell Signaling Technology) overnight incubation, secondary antibody incubation for 1 h, DAB color development, hematoxylin counterstaining, dehydration and mounting, and microscopy.

### Quantitative real-time PCR

Total RNA was extracted from mouse liver tissues or cells with TRIzol Reagent Kit (Lot: DP431, Tiangen Biochemical Technology (Beijing) Co., Ltd.), and the concentration and purity of RNA were examined using a spectrophotometer. cDNA was synthesized from 1 mg of total RNA using a reverse transcriptase kit (Lot: KR106, Tiangen Biochemical Technology (Beijing) Co., Ltd.). Primer sequences outlined in **Table 1** were used to measure and quantify target mRNA levels by the quantitative real-time (RT)-PCR method. The relative mRNA expression levels of STAT3, HIF-1α, Beclin-1, BNIP3, TNF-α, MCP-1, IL-1β, and IL-6 genes were calculated by the 2^-△△Ct^ method after standardization based on the β-actin transcription (Lot: FP206, Tiangen Biochemical Technology (Beijing) Co., Ltd.).

**Table 1:** Primer sequences to measure mRNA levels using quantitative RT-PCR.

Gene (Mus musculus)	Primer	Sequence (5′–3′)	PCR product (bp)
STAT3	Forward	AATCTCAACTTCAGACCCGCCAAC	120
	Reverse	GCTCCACGATCCTCTCCTCCAG	
HIF-1α	Forward	CCACCACAACTGCCACCACTG	141
	Reverse	TGCCACTGTATGCTGATGCCTTAG	
Beclin-1	Forward	TCTGAAACTGGACACGAGCT	162
	Reverse	CCCCGATCAGAGTGAAGCTA	
BNIP3	Forward	CTCCTGGGTAGAACTGCACT	175
	Reverse	ATCTTGTGGTGTCTGGGAGC	
TNF-α	Forward	AGCCTCTTCTCATTCCTGCT	116
	Reverse	CTGATGAGAGGGAGGCCATT	
MCP-1	Forward	TCACCAGCAAGATGATCCCA	117
	Reverse	CAGCACAGACCTCTCTCTTGA	
IL-1β	Forward	TTGAAGAAGAGCCCGTCC	172
	Reverse	CTTATGTTCTGTCCATTGAGG	
IL-6	Forward	GAGACTTCCATCCAGTTGCC	114
	Reverse	CAGGTCTGTTGGGAGTGGTA	
β-Actin	Forward	GGCACAGTCAAGGCTGAGAA	143
	Reverse	ATGGTGGTGAAGACGCCAGTA	

### Western blotting

Mouse liver tissue or experimental cells were dissolved in RIPA buffer for 30 min. The supernatant was collected after being centrifuged at 12,000 rpm and 4°C for 10 min. The total protein concentration was determined using the BCA method. The protein samples were separated by 10% SDS-PAGE and electrotransferred to polyvinylidene fluoride (PVDF) membranes. The PVDF membranes were blocked with 5% non-fat dry milk for 2 h at room temperature and then incubated with the primary antibodies (P-STAT3, STAT3, HIF-1α, p62, BNIP3, Beclin-1, LC3 A/B, and P-NF-κB p65 at 1:2,000 dilution, Lot: 9145S; 9139S; 36169S; 8025S; 3769S;3738S; 12741; 3033, Cell Signaling Technology or Bax, Bcl-2, cleaved caspase-3, and β-actin at 1:3,000 dilution, Lot: ab32503; ab59348; ab214430; ab8227, Abcam) overnight at 4°C. After incubation with the secondary antibody (1:10,000) for 1 h, the bands were visualized by an enhanced chemiluminescence system. Each group experiment was repeated three times. Quantitative analysis was performed with ImageJ software (National Institutes of Health, United States).

### Fluorescence and flow cytometry

After the indicated treatments, cells were fixed with 4% paraformaldehyde and permeabilized in PBS containing 0.1% Triton X-100 (Sigma-Aldrich). Cells were incubated with rabbit anti-LC3 overnight at 4°C. The next day, a fluorescent secondary antibody (1:100) was added and incubated at 4°C for 1 h in the dark. Images were acquired using a fluorescence microscope. Six to eight different fields were randomly selected from each group, and the average LC3 region fluorescence intensity was analyzed and calculated by ImageJ software. After the culture, the cells in each group were collected and washed twice with PBS. According to the operation procedure of the PE-conjugated Annexin-V Apoptosis Detection Kit (Lot: 559763, Becton, Dickinson and Company), antibody incubation and FACSCalibur flow cytometer on-board detection (Becton, Dickinson and Company) were carried out.

### Statistical analysis

GraphPad Prism 5 software was used to analyze the experimental data using a two-tailed Student’s t-test or one-way ANOVA test, and the measurement data were described as mean ± SEM. *p* < 0.05 indicated a statistically significant difference.

## Results

### α-GA improves the pathological changes of the liver and abnormal elevated levels of serum biochemical and inflammatory indexes in NAFLD mice

**Figure 1:**
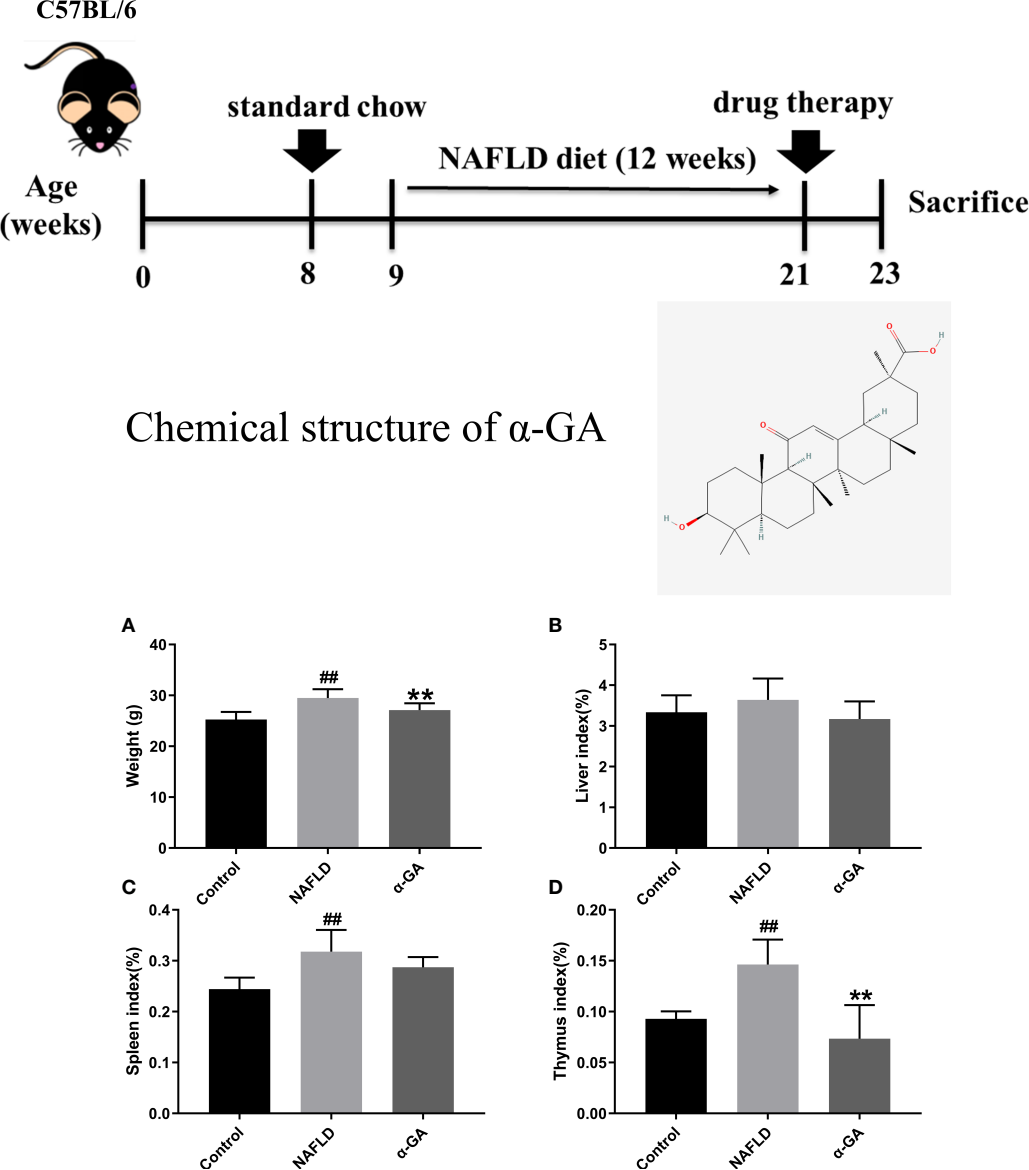
Weight and organ index of mice. **(A)** Body weight. **(B)** Liver index. **(C)** Spleen index. **(D)** Thymus index. mean ± SEM. ^#^*p* < 0.05, ^##^*p* < 0.01, compared with the control group; ^*^p < 0.05, ^**^p < 0.01, compared with the NAFLD group. n = 6/group.

Compared with the control group, the body weight of mice in the NAFLD group increased significantly (*p* < 0.01), and α-GA could significantly inhibit the weight gain (*p* < 0.01). Compared with normal mice, a high-fat and high-sugar diet caused a slight increase in the liver index of NAFLD mice, and α-GA could alleviate this trend. However, there was no significant difference between the control group, NAFLD group, and α-GA group (*p* >0.05). The spleen index and thymus index of the mice in the NAFLD group were significantly higher than those in the control group (*p* < 0.01), indicating that significant immune enhancement and inflammatory response appeared in NAFLD mice. Compared with the NAFLD group, the spleen index in the α-GA group decreased, but there was no statistical significance (*p*>0.05). The intervention of α-GA could effectively reduce the abnormal increase of the thymus index in NAFLD mice (*p* < 0.01). This manifested that GA had certain immunomodulatory and anti-inflammatory effects (**Figure 1**).

**Figure 2:**
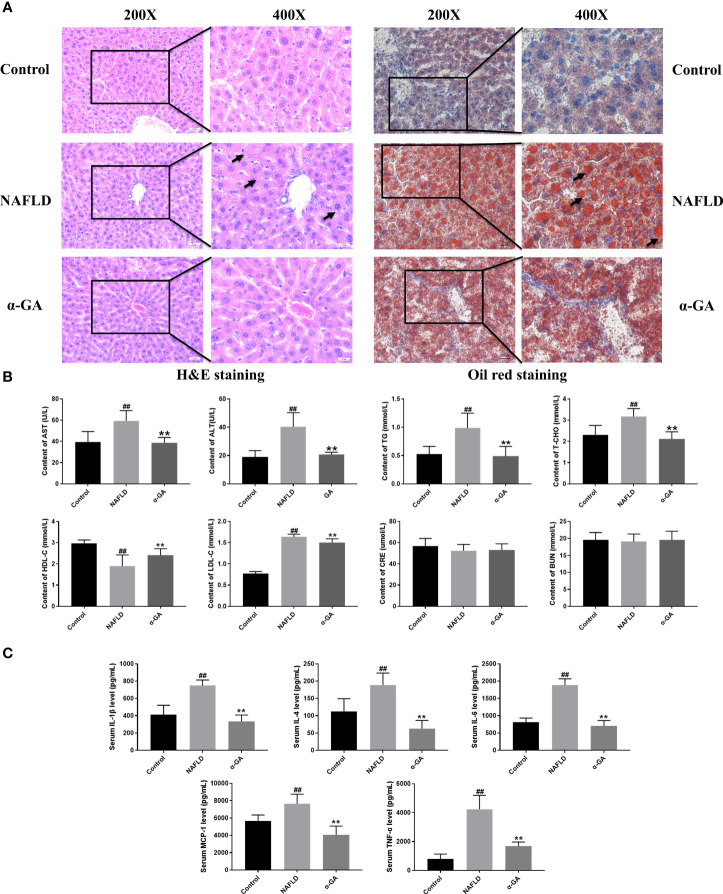
Effects of α-GA on pathological changes of liver and serum biochemical and inflammatory indexes in NAFLD mice. **(A)** H&E staining and Oil Red O staining. **(B)** Serum biochemical indexes. **(C)** Serum inflammatory indexes. mean ± SEM. ^#^*p* < 0.05, ^##^*p* < 0.01, compared with the control group; ^*^p < 0.05, ^**^p < 0.01, compared with the NAFLD group. n = 6/group.

Hepatic tissue H&E and Oil Red O staining confirmed the significant fat deposition in the liver of NAFLD mice. After 2 weeks of intragastric administration of α-GA, hepatic lipid deposition was improved (**Figure 2A**). We found that serum AST, ALT, TG, T-CHO, and LDL-C levels were significantly increased (*p* < 0.01), and the HDL-C level was significantly decreased (*p* < 0.01) in the NAFLD group. After α-GA intervention, those abnormally elevated levels of serum biochemical indexes were significantly improved (*p* < 0.01) and had no significant effect on the renal function indexes of CRE and BUN in mice (*p* > 0.05). α-GA regulated liver lipid metabolism and improved liver cell damage (**Figure 2B**). Long-term liver fat deposition could lead to liver inflammation. Furthermore, it was found that the mice in the NAFLD group had a strong inflammatory response, with the levels of serum inflammatory indicators IL-1β, IL-4, IL-6, MCP-1, and TNF-α significantly increased (*p* < 0.01). α-GA demonstrated a good anti-inflammatory effect (*p* < 0.01) (**Figure 2C**).

### α-GA ameliorates excessive hepatic macrophage infiltration and apoptosis in NAFLD mice

**Figure 3:**
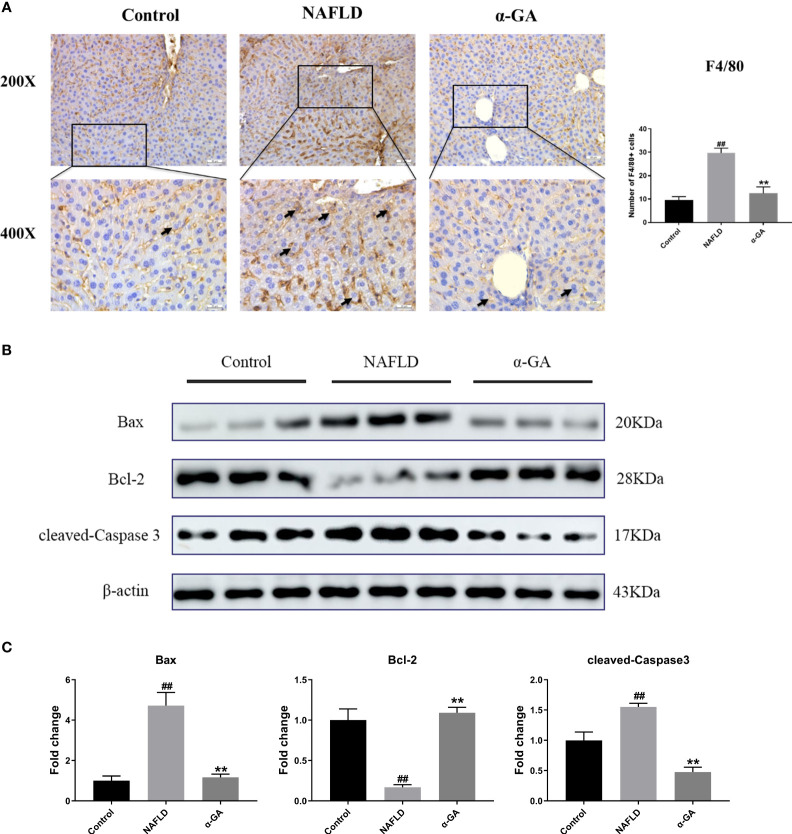
Effects of α-GA on hepatic macrophage infiltration and apoptosis in NAFLD mice. **(A)** Liver sections were subjected to F4/80 immunohistochemistry (black arrows indicate the F4/80-positive cells). The total number of F4/80-positive cells from five high-powered fields was counted per liver section by microscopy. At least three liver sections were included in each group. **(B)** Representative apoptosis-related protein expression bands (Bax, Bcl-2, and cleaved-Caspase 3). **(C)** Quantitative expressional analysis of WB bands. mean ± SEM. ^#^*p* < 0.05, ^##^*p* < 0.01, compared with the control group; ^*^p < 0.05, ^**^p < 0.01, compared with the NAFLD group. n = 6/group.

Previous studies have shown that hepatic macrophages primarily expressing F4/80 are responsible for the aggressiveness of liver injury (33). Infiltration of macrophages into the liver is a hallmark and causes hepatic inflammatory injury (34). To investigate whether α-GA plays a potential role in the regulation of hepatic macrophages in NAFLD mice, we detected the expression of F4/80 in liver tissues. Compared with mice in the control group, the infiltration of macrophages in the liver tissues of the mice in the NAFLD group increased (*p* < 0.01), and the hepatic infiltration of macrophages was significantly decreased after α-GA treatment (*p* < 0.01). Given the important role of macrophages in NAFLD, it was suggested that the therapeutic effects of α-GA on NAFLD may be related to the regulation of mouse macrophages to exert anti-inflammatory immunity (**Figure 3A**). Previous results have also shown that GL can prevent neutrophils and macrophages from infiltrating in liver injury (35). At the same time, we also noticed that α-GA could indeed improve the apoptosis of liver tissue in NAFLD mice, accompanied by an increase in the level of Bcl-2 protein (*p* < 0.01) and a significant decrease in the level of Bax and cleaved-Caspase 3 protein (*p* < 0.01) (**Figures 3B**, **C**).

### α-GA modulates the hepatic STAT3-HIF-1α pathway and ameliorates impaired autophagic flux

**Figure 4:**
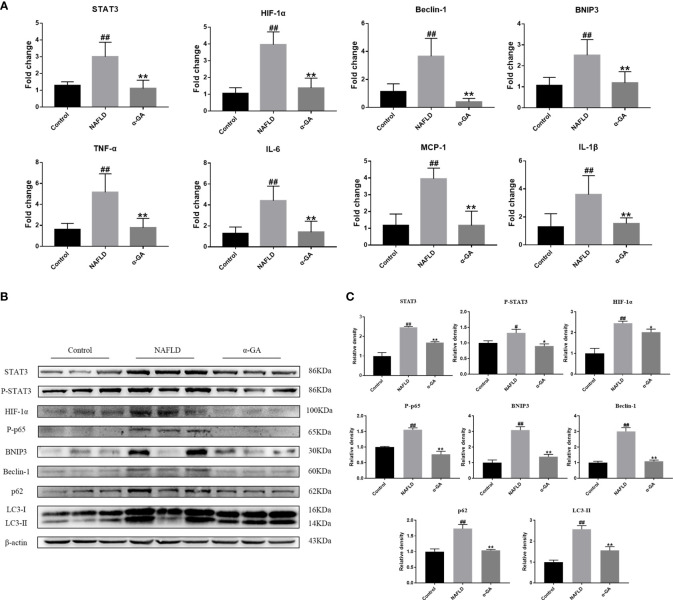
Effects of α-GA on the hepatic STAT3-HIF-1α pathway and autophagic flux. **(A)** The hepatic expression of STAT3-HIF-1α-related genes was measured by RT-PCR. **(B)** Representative apoptosis-related protein expression bands (STAT3, P-STAT3, HIF-1α, P-p65, BNIP3, Beclin-1, p62, and LC3II). **(C)** Quantitative expressional analysis of WB bands. Mean ± SEM. ^#^*p* < 0.05, ^##^*p* < 0.01, compared with the control group; ^*^p < 0.05, ^**^p < 0.01, compared with the NAFLD group. n = 6/group.

As shown in **Figure 4A**, compared with the normal mice, the mRNA expression levels of STAT3, HIF-1α, Beclin-1, BNIP3, TNF-α, MCP-1, IL-1β, and IL-6 in the liver tissue of the NAFLD group mice were significantly increased (*p* < 0.01), and the expression levels of STAT3-HIF-1α pathway-related genes were significantly decreased after α-GA treatment (*p* < 0.01). The protein expression levels of STAT3, P-STAT3, HIF-1α, P-p65 (NF-κB activation marker), BNIP3 (the transcriptional target of HIF-1α), Beclin-1 (the autophagy-inducing protein), p62 (a marker of impaired autophagy flux), and LC3II (a marker of autophagy-inducible) were significantly increased (*p* < 0.05, *p* < 0.01). The increasing trend was significantly improved after α-GA intervention (*p* < 0.05, *p* < 0.01). The protein levels of P-STAT3 and HIF-1α in the liver tissue of NAFLD mice were significantly increased, and the expressions of autophagy markers BNIP3, Beclin-1, p62, and LC3II and the NF-κB activation marker P-p65 were consequently increased, indicating impaired hepatic autophagic flux and excessive inflammation. GA could regulate the STAT3-HIF-1α pathway to improve impaired autophagy flow and excessive inflammatory response in liver tissues of NAFLD mice (**Figures 4B**, **C**).

### α-GA modulates the PA-induced macrophage STAT3-HIF-1α pathway and ameliorates impaired autophagic flux

**Figure 5:**
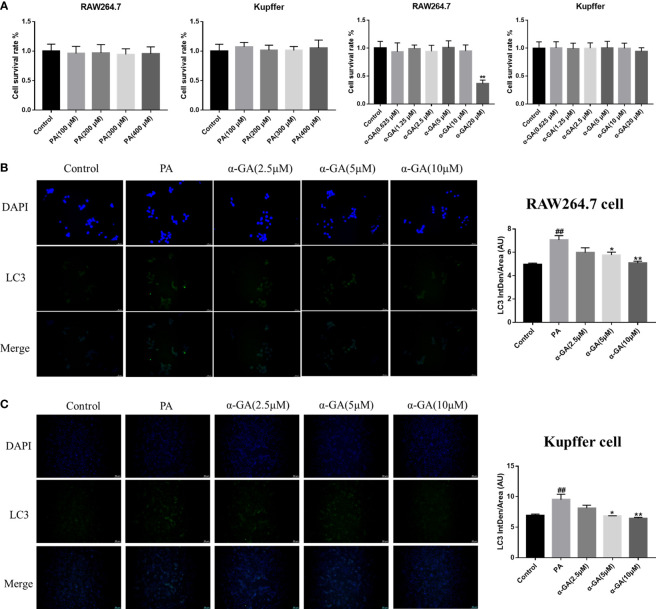
Effects of α-GA on the expression of PA-induced LC3 fluorescent protein in macrophages. **(A)** Effects of PA and α-GA on RAW264.7 and Kupffer cell viability measured by CCK8. **(B)** The detection of LC3 puncta in RAW264.7 cells was performed with anti-LC3 antibody and immunofluorescence staining (left). Quantification of LC3 fluorescence intensity (right). **(C)** The detection of LC3 puncta in Kupffer cells and immunofluorescence staining (left). Quantification of LC3 fluorescence intensity (right). Mean ± SEM. ^#^*p* < 0.05, ^##^*p* < 0.01, compared with the control group; ^*^p < 0.05, ^**^p < 0.01, compared with the NAFLD group. n = 6/group.

Hepatic macrophages play a key role in maintaining the homeostasis of the liver and the whole body through five major functions. These include removal of cellular debris and metabolic waste, maintenance of iron homeostasis, regulation of cholesterol homeostasis, modulation of antimicrobial defenses, and promotion of immune tolerance (36–39). To further elucidate the effect of α-GA on macrophages in the progression of NAFLD, first, the effects of PA and α-GA on the viability of RAW264.7 and Kupffer cells were investigated. The results showed that different concentration gradients of PA (100, 200, 300, 400 μM) had no significant effect on the viability (*p* > 0.05). Different concentrations of α-GA had no significant effect on the cell viability (*p* > 0.05), except that 20 μM had a certain inhibitory effect on RAW264.7 cell (*p* < 0.01) (**Figure 5A**). In order to further investigate the effect of FFAs on macrophages *in vivo*, the same dose (300 μM) of PA was used to induce macrophages in the subsequent experimental design. α-GA was set to low, medium, and high doses of 2.5, 5, and 10 μM, respectively.

After PA induction with/without α-GA intervention in RAW264.7 and Kupffer cells, the changes in the expression of autophagy marker LC3 fluorescent protein were observed. We found that compared with normal cells, the expression of LC3 fluorescent protein was significantly enhanced in PA-induced macrophages (*p* < 0.01), and α-GA could attenuate this enhanced trend in a concentration-dependent manner (**Figures 5B**, **C**).

**Figure 6:**
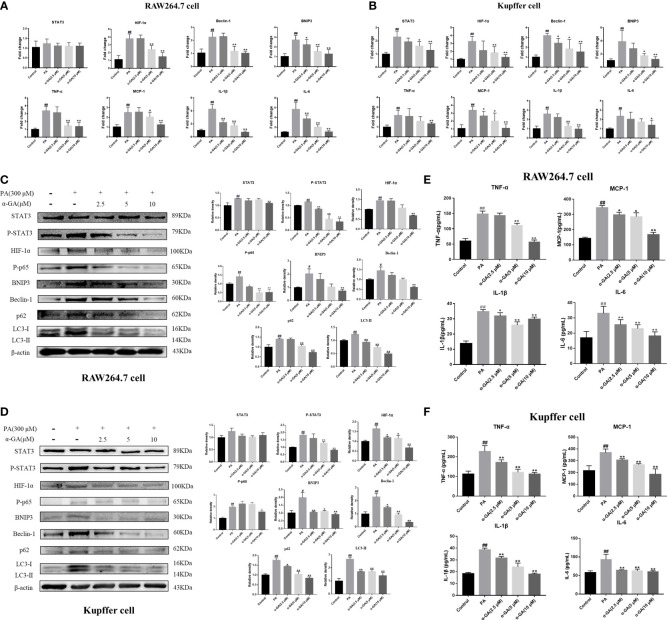
Effects of α-GA on the PA-induced macrophage STAT3-HIF-1α pathway and impaired autophagic flux. **(A, B)** Effects of α-GA on the STAT3-HIF-1α pathway and autophagy gene expression induced by PA in RAW264.7 and Kupffer cells (STAT3, HIF-1α, Beclin-1, BNIP3, TNF-α, MCP-1, IL-1β, and IL-6). **(C, D)** Effects of α-GA on the STAT3-HIF-1α pathway and autophagy protein expression induced by PA in RAW264.7 and Kupffer cells (STAT3, P-STAT3, HIF-1α, P-p65, BNIP3, Beclin-1, p62, and LC3II). Representative apoptosis-related protein expression bands (left). Quantitative expressional analysis of WB bands (right). **(E, F)** Effects of α-GA on the expression of inflammatory cytokines induced by PA in RAW264.7 and Kupffer cells (TNF-α, MCP-1, IL-1β, and IL-6). Mean ± SEM. ^#^*p* < 0.05, ^##^*p* < 0.01, compared with the control group; ^*^p < 0.05, ^**^p < 0.01, compared with the NAFLD group. n = 6/group.

To clarify whether the enhanced expression of LC3 fluorescent protein represents the activation of autophagy in macrophages, the study further explored the relationship between the STAT3-HIF-1α pathway, autophagy flux, and the pharmacological mechanism of α-GA in PA-induced macrophages. The levels of the STAT3-HIF-1α pathway and autophagy-related genes and proteins were significantly upregulated after PA induction and decreased in a concentration-dependent manner after α-GA intervention (**Figures 6A**–**D**). In addition, α-GA could improve the abnormal increase in the levels of inflammatory cytokines such as TNF-α, MCP-1, IL-1β, and IL-6 in the cell supernatant induced by PA (**Figures 6E**, **F**).

**Figure 7:**
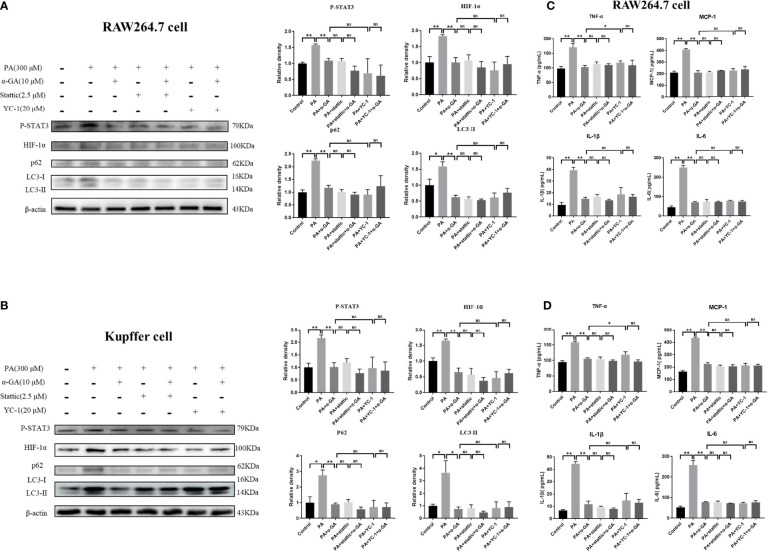
Effects of α-GA on PA-induced impaired autophagic flux in macrophages by regulating STAT3 and HIF-1α. **(A, B)** The protein expression of P-STAT3, HIF-1α, p62, and LC3II in RAW264.7 and Kupffer cells. Representative apoptosis-related protein expression bands (left). Quantitative expressional analysis of WB bands (right). **(C, D)** Effects of α-GA on the expression of inflammatory cytokines in RAW264.7 and Kupffer cells (TNF-α, MCP-1, IL-1β, and IL-6). Mean ± SEM. *p < 0.05, **p < 0.01, ns (no significance), p>0.05. n = 6/group.

Furthermore, the inhibitors were used to investigate whether α-GA ameliorated the PA-induced impairment of autophagic flux in macrophages through the STAT3-HIF-1α pathway. PA could significantly increase the protein levels of P-STAT3, HIF-1α, p62, and LC3II in RAW264.7 cells (*p* < 0.05, *p* < 0.01), and α-GA treatment significantly decreased the expression of these proteins (*p* < 0.01). Compared with the PA group, the protein levels of P-STAT3, HIF-1α, p62, and LC3II were also significantly reduced after the intervention of the inhibitors Stattic and YC-1, indicating that the PA-induced impaired autophagic flux in macrophages was associated with enhanced STAT3 phosphorylation and HIF-1α hyperactivation. At the same time, there were no significant differences in the protein levels of P-STAT3, HIF-1α, p62, and LC3II between the inhibitor-only intervention group and α-GA with/without Stattic or YC-1 (*p* > 0.05). Similar findings were also found in the levels of inflammatory cytokines in the cell supernatant. These results suggested that α-GA had similar mechanisms of action to Stattic and YC-1. It could improve the PA-induced impairment of autophagic flux in macrophages and reduce the excessive production of inflammatory cytokines, possibly by inhibiting STAT3 phosphorylation and HIF-1α excessive activation (**Figure 7**).

### α-GA ameliorates PA-induced blockade of autophagosome–lysosome fusion in macrophages and inhibits hepatocyte apoptosis

**Figure 8:**
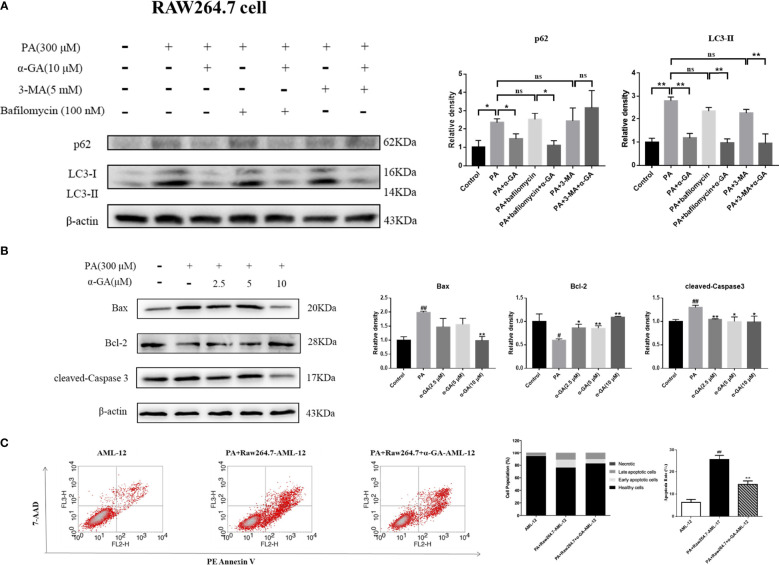
Effects of α-GA on hepatocyte apoptosis by regulating autophagic flux in RAW264.7 cells. **(A)** The protein expression of p62 and LC3II. The RAW264.7 cells were treated with bafilomycin A1 (interferes with the fusion of autophagosomes and lysosomes) and 3-MA (blocks autophagosome formation), with or without α-GA. Representative apoptosis-related protein expression bands (left). Quantitative expressional analysis of WB bands (right). *p < 0.05, **p < 0.01, ns (no significance), p>0.05. n = 6/group. **(B)** Effects of specifically treated RAW264.7 cell supernatants on the proteins associated with apoptosis in AML-12 cells (Bax, Bcl-2, and cleaved-Caspase 3). Representative apoptosis-related protein expression bands (left). Quantitative expressional analysis of WB bands (right). **(C)** The apoptosis rate of AML-12 cells was measured by flow cytometry. Mean ± SEM. ^#^*p* < 0.05, ^##^*p* < 0.01, compared with the control group; ^*^p < 0.05, ^**^p < 0.01, compared with the NAFLD group. n = 6/group.

Autophagy of macrophages can significantly affect pathogen-recognition receptors (40). As ligands of Toll-like receptors, saturated fatty acids are associated with the pathogenesis of NAFLD. In order to further clarify the mechanism of PA on the impaired autophagic flux of macrophages and the regulatory effect of α-GA, bafilomycin A1 (interferes with the fusion of autophagosomes and lysosomes) and 3-MA (blocks autophagosome formation) were applied. In RAW264.7 cells, bafilomycin A1 or 3-MA combined with PA induction had no significant difference in p62 and LC3II protein levels compared with a single PA induction (*p* > 0.05), indicating that the PA-induced impairment of autophagic flux may be related to the blocking of autophagosomes formation and the fusion of autophagosomes and lysosomes. Compared with the bafilomycin A1 group, α-GA supplement could significantly reduce the p62 and LC3II protein levels (*p* < 0.05, *p* < 0.01). However, compared with the 3-MA group, α-GA supplement significantly decreased the LC3 II protein level (*p* < 0.01) but had no significant effect on the P62 protein level (*p* > 0.05), suggesting that α-GA regulated the above two autophagy processes but mainly improved the blockade of the autophagosome–lysosome fusion (**Figure 8A**).

**Figure 9:**
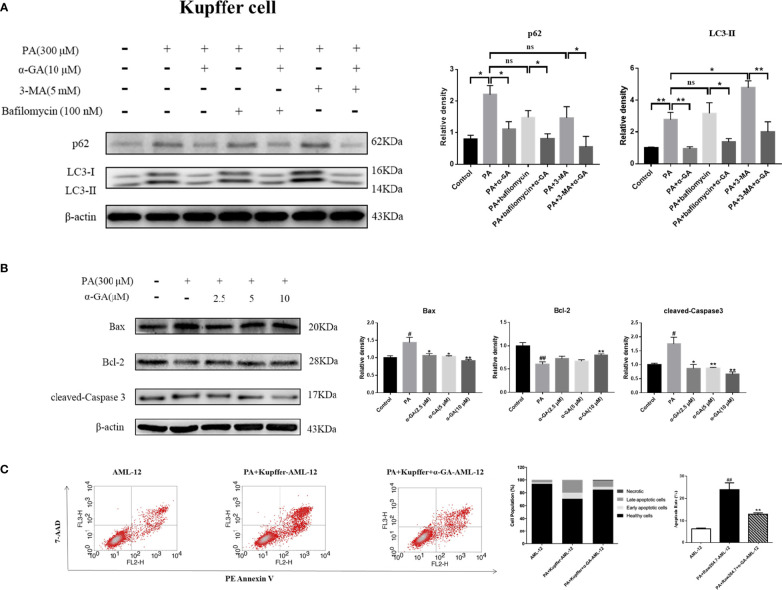
Effects of α-GA on hepatocyte apoptosis by regulating autophagic flux in Kupffer cells. **(A)** The protein expression of p62 and LC3II. The Kupffer cells were treated with bafilomycin A1 (interferes with the fusion of autophagosomes and lysosomes) and 3-MA (blocks autophagosome formation), with or without α-GA. Representative apoptosis-related protein expression bands (left). Quantitative expressional analysis of WB bands (right). *p < 0.05, **p < 0.01, ns (no significance), p>0.05. n = 6/group. **(B)** Effects of specific treated Kupffer cells supernatants on the proteins associated with apoptosis in AML-12 cells (Bax, Bcl-2, and cleaved-caspase 3). Representative apoptosis-related protein expression bands (left). Quantitative expressional analysis of WB bands (right). **(C)** The apoptosis rate of AML-12 cells was measured by flow cytometry. mean ± SEM. ^#^*p* < 0.05, ^##^*p* < 0.01, compared with the control group; ^*^p < 0.05, ^**^p < 0.01, compared with the NAFLD group. n = 6/group.

In Kupffer cells, there was no significant difference in p62 protein level between bafilomycin A1 or 3-MA combined with PA and PA alone (*p* > 0.05). Compared with PA alone, there was no significant difference in the LC3 II protein level in bafilomycin A1 combined with PA induction (*p* > 0.05), but there was an increase in 3-MA combined with PA induction (*p* < 0.05). These results indicated that the PA-induced impaired autophagy flux in Kupffer cells was mainly related to the blocking of the fusion of autophagosome and lysosome. Overall, α-GA could effectively ameliorate the PA-induced impairment of autophagic flux in macrophages, and its mechanism was mainly related to the promotion of interference with the fusion of autophagosomes and lysosomes (**Figure 9A**).

What is the effect of α-GA on hepatocytes by improving PA-induced impairment to macrophage autophagy flux? After intervention for 24 h of normal medium, PA, and PA plus α-GA (2.5, 5, and 10 μM), the supernatant of macrophages was then extracted after 24 h of starvation treatment, which corresponded to the configuration of AML-12 hepatocyte medium. The supernatant of each group was mixed with normal AML-12 cell culture medium (1:1) and added to the cells for 24 h of intervention culture, and then the apoptosis was investigated. Macrophage supernatant induced by PA could lead to excessive apoptosis of hepatocytes; specifically, Bax and cleaved-caspase 3 protein levels were significantly increased and Bcl-2 protein levels were significantly decreased, which were significantly reversed after intervention with different doses of α-GA (**Figure 8B**, **Figure 9B**). Further, we analyzed the effect of two types of treated macrophage supernatants on hepatocyte apoptosis rate by flow cytometry. The specifically treated RAW264.7 cell and Kupffer cell supernatants caused 25.56% and 23.91% of hepatocyte apoptosis, respectively, while the supernatants treated with α-GA (10 μM) had significantly reduced apoptotic rates of 14.41% and 12.88% (**Figure 8C**, **Figure 9C**).

## Discussion

Compared with other organs, the liver is rich in macrophages, and the sources include Kupffer and myeloid-derived monocytes/macrophages from the fetal yolk sac. Liver Kupffer cells and monocyte-derived macrophages integrate signals from the gut–liver axis, overnutrition, systemic low-grade inflammation, and steatosis, driving the progression of NAFLD and even liver fibrosis (10, 41). Clinically, NAFLD often has complications of obstructive sleep apnea, leading to periodic hypoxia. Obstructive sleep apnea and nocturnal hypoxia are important triggers for the progression of NAFLD (42). Intermittent hypoxia has been shown to cause tissue hypoxia and may contribute to abnormal hepatic lipid metabolism, mitochondrial dysfunction, oxidative stress, insulin resistance, inflammation, and hyperactivation of the sympathetic nervous system, contributing to the progression of NAFLD (43). HIF has been extensively studied in cancer, but there is evidence that this pathway has an important role in controlling metabolism and affecting NAFLD and Mets (44). Autophagy is an important regulatory mechanism to maintain cellular homeostasis and plays a key role in the occurrence and development of NAFLD. Impaired autophagy (decreased autophagy or blocked autophagic flux) leads to cell damage and death and promotes liver disease progression, a phenomenon well established in NAFLD livers, hepatocytes, endothelial cells, and hepatic stellate cells (45–48). Growing evidence from animal and clinical studies suggests that targeting autophagy of macrophages may be an effective therapeutic strategy for NAFLD and related Mets (49).

Research evidence shows that primary bone marrow-derived macrophages and peritoneal macrophage autophagic flux levels are significantly reduced in mice fed a high-fat diet, suggesting a general impairment of macrophage autophagy in obese mice. Excessive lipid accumulation reduces the level of autophagy, and impaired macrophage autophagy may promote the activation of innate immunity leading to obesity, mainly manifested in abnormal polarization of macrophages (increased M1 type and decreased M2 type). This suggests that autophagy has an important regulatory function in macrophage polarization and can downregulate inflammatory responses. Impaired macrophage autophagy may underlie the body’s inflammatory state, which in turn leads to the progression of liver inflammation and liver injury (50).

**Figure 10:**
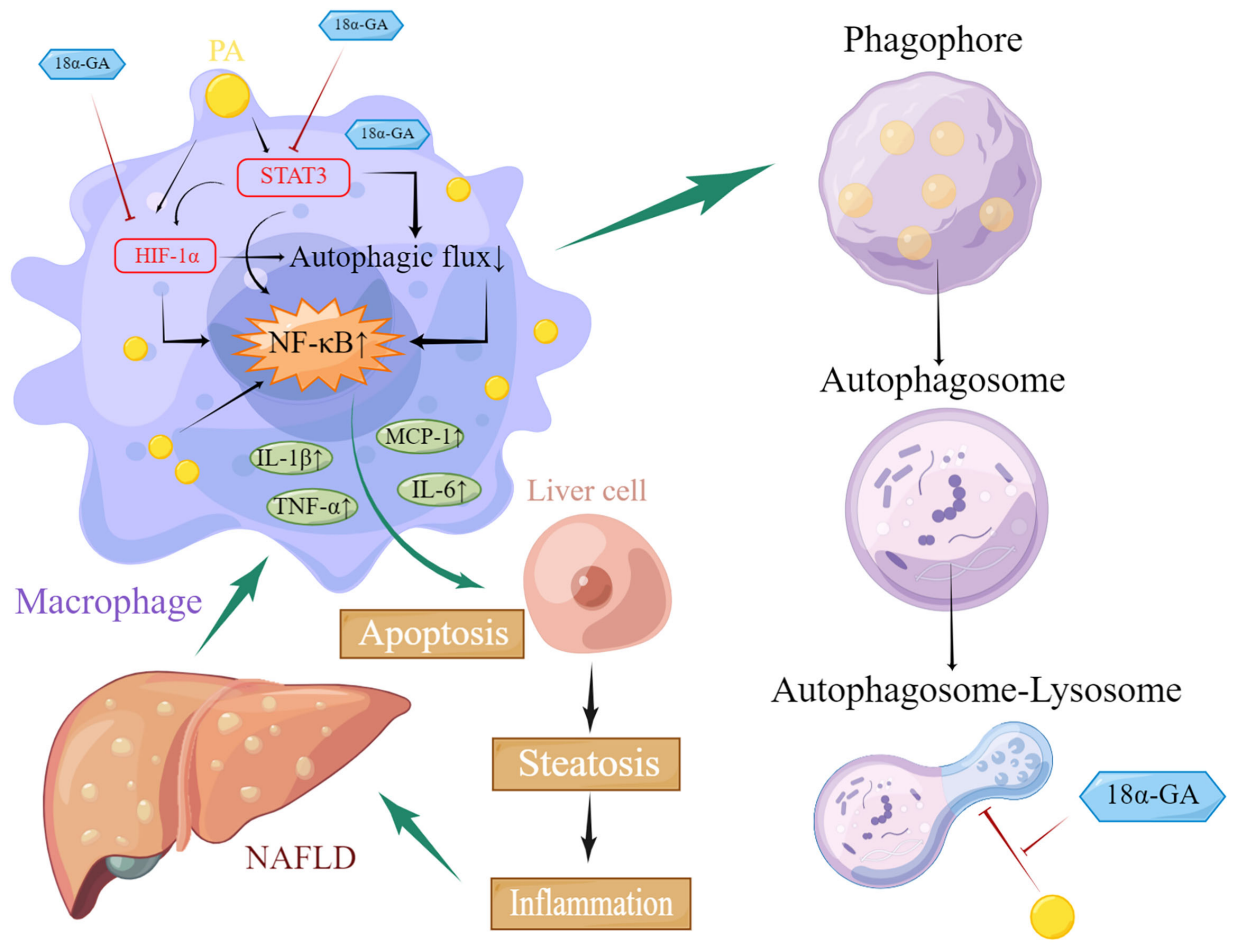
Schematic diagram of glycyrrhetinic acid (α-GA) regulating impaired macrophage autophagic flux in treatment of non-alcoholic fatty liver disease (NAFLD). Excess lipids (palmitic acid, PA) accumulate in macrophages, causing STAT3 phosphorylation and HIF-1α hyperactivation, impairing its autophagic flux, mainly hindering the fusion of autophagosomes and lysosomes. The subsequent enhancement of autoimmunity especially the overactivation of NF-κB led to the excessive release of inflammatory cytokines including TNF-α, MCP-1, IL-1β, and IL-6, which caused excessive apoptosis of hepatocytes and impaired functions of liver tissues through paracrine, thereby triggering NAFLD. α-GA can inhibit the impaired autophagic flux and excessive production of inflammatory cytokines in macrophages caused by NAFLD by regulating the overactivation of the STAT3-HIF-1α pathway in macrophages and improving the excessive apoptosis of hepatocytes, thereby exerting the therapeutic effect of NAFLD.

Our results suggested that GA could improve liver pathological changes and lipid metabolism abnormalities in NAFLD mice. GA reduced excessive infiltration of macrophages in the liver and excessive apoptosis of liver cells caused by a high-fat and high-sugar diet. Further investigation found that GA could improve the impaired autophagy of macrophages, which was related to the regulation of STA3-HIF-1α. (**Figure 10**). STAT3 is involved in the whole process of assembly and maturation from autophagosomes, and the activation of STAT3 transcriptional activity mainly depends on the phosphorylation of a single tyrosine residue Tyr705, which can be controlled by receptor tyrosine kinases such as MET, KDR, and EGFR, or non-receptor tyrosine kinases. It is directly catalyzed by tyrosine kinases such as JAKs, and phosphorylation at Ser727 of serine determines its maximal activation state. Nuclear STAT3 binds to specific DNA components to transcriptionally activate or repress target genes, such as BCL2, BECN1, PIK3C3, CTSB, CTSL, PIK3R1/p55α, PIK3R1/p50α, and MIR17HG and HIF-1α and BNIP3, depending on the cellular environment or stimulus, inhibit or stimulate autophagy. STAT3 monomers can also be transferred to mitochondria, interact with electron transport chain complexes I and II, inhibit the production of reactive oxygen species, reduce HIF-1α activity, and inhibit autophagy (51). The regulation of HIF-1α by STAT3 is divided into two aspects. On the one hand, STAT3 transcriptionally upregulates HIF-1α gene expression. On the other hand, STAT3 interacts with the C-terminal domain of HIF-1α and stabilizes the protein through von Hippel–Lindau-mediated ubiquitination. In NASH patients as well as mouse liver and cell models, the expression of STAT3 and p-STAT3 is increased, and autophagy is inhibited. Downregulation of STAT3 expression can activate autophagy and inhibit the inflammatory response of NASH (52, 53). In NASH, FFAs are important lipotoxic mediators that lead to cellular damage and induce liver damage through endoplasmic reticulum stress. Hypoxia enhances PA-induced activation of the pro-inflammatory state of human macrophages (54). Previous studies have also shown that in hepatic steatosis, HIF-1α and autophagy are involved in FFA-induced cellular stress in hepatocytes (55, 56). Recent results further confirmed that α-GA inhibits STAT3 Tyr705 phosphorylation by increasing protein tyrosine phosphatase 1 and 2 expressions and inhibits TGF-β-triggered hepatocellular carcinoma invasion and metastasis *in vivo* and *in vitro (* 57). In addition, GA inhibits phosphorylation at Ser727 of STAT3 (58). This is consistent with previous reports that GA inhibits STAT3 phosphorylation in the treatment of colon cancer (59), psoriasis (60), and hepatocellular carcinoma (57). A recent study in China shows that GL reduces the expression level of HIF-1α, inhibits the release of inflammatory cytokines IL-6 and TNF-α, and plays protective roles in acute lung injury.

Due to their central position in the hepatic microenvironment, their long cytoplasmic protrusions, and the high density of pattern recognition receptors on their surface, hepatic macrophages act as initial sensors of liver injury (61, 62). The activation of hepatic macrophages and subsequent secretion of pro-inflammatory mediators (dependent on NLRP3 and NF-κB activation) lead to increased lipid accumulation and damage in hepatocytes, which are key events in NAFLD development and progression (63). During NAFLD/NASH, reactive oxygen species and damage-associated molecular patterns released from injured hepatocytes undergoing apoptosis or necrosis trigger macrophages secrete a variety of chemokines to recruit monocytes and other leukocytes, leading to insulin resistance and oversecretion of proinflammatory and chemokines such as TNF-α, IL-6, IL-1β, and MCP-1 (64). Hepatic macrophages are a major source of MCP-1, which will further recruit CCR2^+^ monocytes to diseased livers (65). Contributions of macrophage-derived cytokines (TNF-α, IFN-γ, and IL-1β), chemokines (MCP-1, IL-6), and reactive oxygen/nitrogen species lead to hepatocyte death and recruitment of additional immune cells that booster liver damage. Macrophages switch their functional characteristics from pro-inflammatory to anti-inflammatory, thereby promoting a resolution response to liver injury (66, 67). Autophagy is a lysosomal degradation pathway of cellular components and exhibits anti-inflammatory properties in macrophages. Studies have shown that macrophage autophagy can be used as a novel anti-inflammatory pathway to regulate NAFLD/NASH and liver fibrosis (68–71). GL/GA has been shown to play a key role in metabolic diseases, cancers, and respiratory diseases through the regulation of inflammatory signaling pathways such as NLRP3 and NF-κB (27, 72, 73). Our results also showed that GA regulated the STAT3-HIF-1α pathway to inhibit the phosphorylation of NF-κB. In the early stage of NAFLD, hepatic macrophages secrete MCP-1 to recruit monocyte-derived macrophages that promote inflammation and fibrosis. Meanwhile, impaired autophagy of hepatic macrophages leads to the release of pro-inflammatory cytokines and chemokines (TNF-α, IL-6, and IL-1β), leading to excessive apoptosis of liver cells and activation of hepatic stellate cells, which drives the progress of NAFLD/NASH (62).

## Conclusion

In all, this study further demonstrated that α-GA could regulate the STAT3-HIF-1α pathway of macrophages, ameliorate the impaired autophagy flux, and reduce excessive production of inflammatory cytokines to improve the excessive apoptosis of liver cells, thus playing a therapeutic role in NAFLD.

## Data availability statement

The original contributions presented in the study are included in the article/supplementary material. Further inquiries can be directed to the corresponding authors.

## Ethics statement

The animal study was reviewed and approved by the Experimental animal ethics committee of the Institute of Tianjin University of Traditional Chinese Medicine. Mouse experimental Ethics: TCM-LAEC2021224.

## Author contributions

YF, LY, and YB conceived and designed the experiments; YF, WD, YW, SZ, and XZ performed the experiments; YF, WD, YY, and RC analyzed the data; YF, YW, LY, and YB wrote the manuscript. All authors have read and approved the final manuscript.

## Funding

This work was supported by the National Key R&D Program of China (2018YFC1706506), 2019 Graduate Innovation Fund of Tianjin University of Traditional Chinese Medicine School of Integrated Traditional Chinese and Western Medicine (ZXYCXLX201907), and 2020 Tianjin Postgraduate Research and Innovation Project (2020YJSB189 and YJSKC-20201003).

## Acknowledgments

We are very grateful for the mice that were sacrificed in this experiment.

## Conflict of interest

The authors declare that the research was conducted in the absence of any commercial or financial relationships that could be construed as a potential conflict of interest.

## Publisher’s note

All claims expressed in this article are solely those of the authors and do not necessarily represent those of their affiliated organizations, or those of the publisher, the editors and the reviewers. Any product that may be evaluated in this article, or claim that may be made by its manufacturer, is not guaranteed or endorsed by the publisher.
